# Targeting Triple-Negative Breast Cancer by the Phytopolyphenol Carnosol: ROS-Dependent Mechanisms

**DOI:** 10.3390/antiox12071349

**Published:** 2023-06-27

**Authors:** Halima Alsamri, Yusra Al Dhaheri, Rabah Iratni

**Affiliations:** 1 General Requirement Department, Fatima College of Health Sciences, Al Ain P.O. Box 24162, United Arab Emirates; halima.alsamri@fchs.ac.ae; 2 Department of Biology, College of Science, United Arab Emirates University, Al Ain P.O. Box 15551, United Arab Emirates; yusra.aldhaheri@uaeu.ac.ae

**Keywords:** oxidative stress, anticancer, TNBC, carnosol, unfolded protein response, targeted protein degradation, apoptosis, autophagy

## Abstract

Triple-negative breast cancer (TNBC), which lacks the expression of the three hormone receptors (i.e., estrogen receptor, progesterone receptor, and human epidermal growth factor receptor), is characterized by a high proliferative index, high invasiveness, poor prognosis, early relapse, and a tendency to be present in advanced stages. These characteristics rank TNBC among the most aggressive and lethal forms of breast cancer. The lack of the three receptors renders conventional hormonal therapy ineffective against TNBC. Moreover, there are no clinically approved therapies that specifically target TNBC, and the currently used chemotherapeutic agents, such as cisplatin, taxanes, and other platinum compounds, have a limited clinical effect and develop chemoresistance over time. Phytochemicals have shown efficacy against several types of cancer, including TNBC, by targeting several pathways involved in cancer development and progression. In this review, we focus on one phytochemical carnosol, a natural polyphenolic terpenoid with strong anti-TNBC effects and its ROS-dependent molecular mechanisms of action. We discuss how carnosol targets key pathways and proteins regulating the cell cycle, growth, epigenetic regulators, invasion, and metastasis of TNBC. This review identifies carnosol as a potential novel targeting protein degradation molecule.

## 1. Introduction

Over the past five decades, deaths from heart disease, stroke, and pneumonia have dropped because of the development of treatment and preventive approaches based on a profound understanding of their risk factors and pathogenesis. However, during the same period, cancer mortality has remained relatively unchanged [1]. We are at a turning point in history as cancer-related deaths currently exceed those from cardiovascular diseases [2,3,4,5].

In 2020, breast cancer was the leading cause of death among women globally. Breast cancer remains the fifth most common cancer in terms of incidence and mortality, accounting for 11.7% of total cancer incidence and 6.9% of total cancer-related deaths [6,7]. Breast cancer is a heterogeneous disease, which makes it challenging to diagnose and treat [8]. Currently, breast cancer is classified based on the proliferation index (Ki67) and expression of hormone receptors, namely estrogen receptor (ER), progesterone receptor (PR), and human epidermal growth factor receptor-2 (HER2). Accordingly, there are four molecular subtypes of breast cancer: luminal A (ER^+^/PR^+^/Ki67 low < 14% or Ki67 intermediate 14–19%); luminal B (ER^+^/PR^+^/HER2^−/+^/Ki67 intermediate 14–19% or Ki67 high > 20%); basal-like or triple-negative (ER^−^/PR^−^/HER2^−^/Ki67); and HER2 overexpressing (ER^−^/PR^−^/HER2^+^/Ki67) [9,10,11,12].

The triple-negative breast cancer (TNBC) subtype accounts for approximately 15–20% of all breast cancer cases [13]. TNBC frequently occurs in younger women (<50 years) and is more prevalent in women of Hispanic and African American origin [14]. TNBC exhibits increased proliferation markers, mitotic activity, high-grade nuclear atypia, scant stromal content, a high nuclear-cytoplasmic ratio, central necrosis, multiple apoptotic cells, a high invasive capacity, and stromal lymphocytic infiltration [15,16]. Additionally, TNBC is characterized by a rapid growth rate, a higher grade compared to other breast cancer subtypes, lymph node progression, and metastasis to other organs, mostly the lungs and brain [17]. TNBC is also reported to be very aggressive and consistently has a poor prognosis, a high reoccurrence rate, and shorter survival [8,9,18,19]. The lack of expression of the three hormonal receptors renders TNBC unresponsive to conventional targeted hormonal therapies (e.g., tamoxifen, aromatase inhibitors) and HER2-directed therapies (e.g., trastuzumab) [20]. The current treatment of TNBC mainly relies on chemotherapy; however, treatment resistance and metastasis still occur [21].

The currently used and approved chemotherapeutic agents for TNBC include cisplatin, anthracycline, taxanes, paclitaxel, tamoxifen, and other platinum compounds [22,23,24]. However, in addition to other conventional therapies, these chemotherapeutic approaches are associated with severe side effects and the development of chemoresistance over time [24]. Identifying a novel anticancer drug for the treatment of TNBC is considered a relatively expensive approach. Currently, cancer research is oriented toward testing plant-derived compounds (i.e., phytochemicals) as an anticancer treatment approach. Plant-derived compounds are convenient to use, are cost-effective, exhibit less toxicity, are generally more effective, and are associated with fewer side effects compared to conventional chemotherapeutic agents, making them a promising option for treating breast cancer, more specifically TNBC.

Several phytochemicals have been reported to display anticancer activity against cancer, including TNBC, such as resveratrol, indole-3-carbino, fisetin, 6-gingerol, curcumin, capsaicin, quercetin, ursolic acid, ailanthone, and cordycepin [25,26,27,28,29,30,31]. These phytochemicals reportedly target several molecular pathways implicated in carcinogenesis, including apoptosis, proliferation, inflammation, invasion, metastasis, angiogenesis, and chemoresistance [25,26,27,28,29,30,31]. Multiple cellular signaling pathways implicated in tumor development and metastasis, such as TNF-α, Notch, Wnt-β, TGF-β, Ras, EGFR, INF-γ, hedgehog, TLR/NF-kB/NLR, PLK, CCNB1, PI3K/AKT, IL-1β, RANKL, SLUG, TWIST1, SNAIL1, and ZEB1, have been modulated by phytochemicals [28,29,30,31,32].

Carnosol, a polyphenolic phytochemical of the terpenoid class, was reported to exert a wide range of biological effects, including anti-inflammatory [33], antioxidant [34], neuroprotective [35], antimicrobial [36], and anticancer effects [28,37,38]. Carnosol reportedly exerts its anticancer effect by modulating various signaling pathways involved in cellular processes, including apoptosis (Bax/Bcl2/caspases), survival and proliferation (Akt/mTOR/MAPK), and transcription factors such as NF-κB and STAT3-6, among others [38,39,40,41]. In the current review, we summarize the recent findings reporting the anti-cancer effect of carnosol against TNBC along with its molecular mechanisms of action.

## 2. Carnosol: Sources, Chemistry, and Structural Characterization

**Figure 1:**
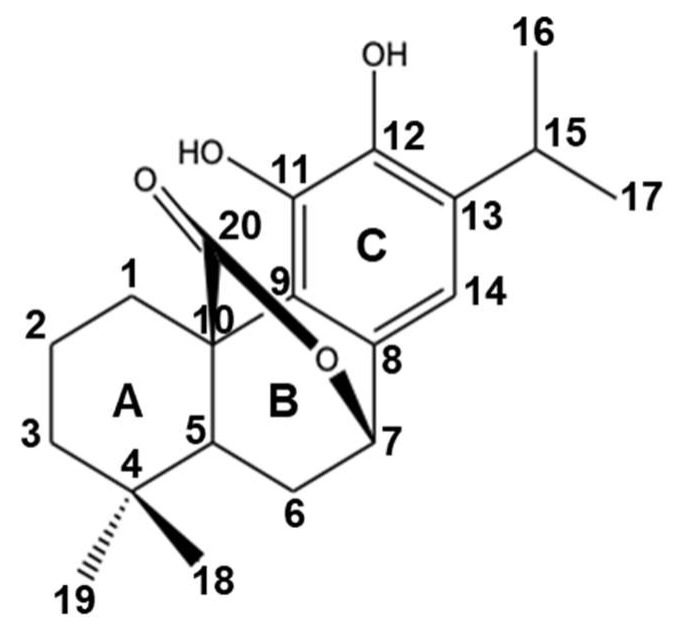
Chemical structure of carnosol.

Rosemary and sage are Mediterranean herbs used for culinary purposes and have been known to contain various bioactive compounds, including polyphenols such as carnosol, carnosic acid, rosmanol, and rosmarinic acid. Carnosol was first isolated from sage (*Salvia carnosa*) in 1942, and its chemical structure (C_20_H_28_O_4_) (Figure 1) was characterized by Brieskorn et al. in 1964 [42]. Carnosol is an ortho-diphenolic diterpene with an abietane carbon skeleton with hydroxyl groups at positions C-11 and C-12 and a lactone moiety across the B ring and is considered to be a byproduct of the oxidative degradation of carnosic acid [43,44]. Carnosol is soluble in various organic solvents, such as dimethyl sulfoxide (250 mg/mL), ethanol (8 mg/mL), and dimethyl formamide (35 mg/mL) [43]. In recent years, carnosol has received increasing attention for its various health-promoting properties.

## 3. Safety and Toxicological Studies on Carnosol

Toxicological studies are essential in the development of novel therapeutic drugs for their potential clinical use. Several animal studies have suggested that the daily oral consumption of carnosol is safe. Johnson et al. reported that carnosol at a dose of 30 mg/kg was well tolerated in mice [37]. Similarly, Sprague–Dawley rats fed with an AIN-76A diet supplemented with 1% carnosol for two weeks showed no changes in body weight [45]. Importantly, the European safety authority has recognized carnosol-enriched rosemary extracts as “Generally Recognized as Safe” (GRAS). In a study testing the toxicity of several rosemary extracts, rats were fed rosemary extracts for 90 days, and the no-observed-adverse-effect level (NOAEL) values were reported to range between 140 and 400 mg extract/kg body weight/day equivalent to 20–60 mg/kg body weight/day of carnosol and carnosic acid [46]. In a more recent study, Phipps et al. investigated the toxicity of carnosol and carnosic acid by feeding male and female mice rosemary extract for 90 days and reported no adverse side effects even at a high dose of 195 mg/kg body weight/day, which is equivalent to 64 mg/kg body weight/day of carnosol and carnosic acid [47]. 

## 4. Carnosol as a Potent ROS Generator in Cancer Cells

Reactive oxygen species (ROS), such as ^1^O_2_, O_2_−, H_2_O_2_, OH•, and HOCl, are byproducts of several cellular processes that can act as second messengers for some important signaling pathways that regulate various cellular processes, including gene expression in both normal and cancer cells [48,49]. Although low to moderate levels of ROS were reported to activate cancer cell proliferation, drug resistance, migration, invasion, and angiogenesis, high concentrations of ROS, on the other hand, induce severe cellular damage, resulting in cell cycle arrest and activation of programmed cell death in cancer cells [48,49]. Indeed, increasing evidence suggests that anticancer drugs that increase intracellular ROS induce cell death in various types of cancer. Thus, anticancer strategies based on the induction of ROS are increasingly considered a promising approach for the treatment of cancer.

Although carnosol was previously described as an antioxidant phytopolyphenol, it was reported to be a potent generator of ROS in MDA-MB-231 and Hs578T TNBC cells [38,50]. Indeed, carnosol induced a concentration-dependent accumulation of ROS, which was detectable as early as 1 h post-treatment and preceded cell death that occurred 24 h post-treatment [38]. In agreement with these findings, carnosol was also reported to induce ROS production in other cancer cells, such as colon (HCT116) [51] and osteosarcoma (MG-63) [52] cells. Inhibition of ROS by the ROS scavengers tiron or N-acetylcysteine (NAC) abrogated carnosol-induced cell death in MDA-MB-231 [38,53], HCT116 [51], and MG-63 [52] cells, thus strongly suggesting that the anticancer activity of carnosol might be mediated mainly through ROS production.

## 5. Anticancer Activities of Carnosol against TNBC

Despite the large number of studies reporting the anticancer activity of carnosol, only a few reports investigated its activity against TNBC. Interestingly, carnosol was shown to inhibit TNBC by targeting diverse pathways involved in proliferation, growth, migration, invasion, and epigenetic regulation. Table 1 summarizes the major targets of carnosol in TNBC.

### 5.1. Carnosol Decreases Cellular Viability and Induces G2 Arrest in TNBC

Aberrant cell cycle progression leading to uncontrolled cellular proliferation is a characteristic of cancer cells. These result from mutations in upstream signaling pathways or in genes encoding cell cycle proteins. Cyclin-dependent kinases (CDKs) are known to be aberrantly activated in many cancers [57]. Therefore, inhibitors of CDKs and cell cycle regulators are attractive targets in cancer therapy. Most of the currently used anticancer drugs, such as tamoxifen and methotrexate [58], limit tumor progression by hindering one or more cell cycle phases and their transition points, thereby attenuating cancer cell proliferation. Cell cycle arrest by these drugs may also be associated with the induction of apoptosis, a type of programmed cell death [59]. Several studies reported that the anticancer effects of carnosol are mediated via the inhibition of cancer cell proliferation either via cell cycle arrest and/or induction of cell death [37,41,60]. Our team was one of the first to explore the anticancer activity of carnosol against TNBC [38]. Carnosol was reported to significantly reduce the cellular viability, in a concentration- and time-dependent manner, of various TNBC cell lines, including MDA-MB-231 [38,53,54,55], MDA-MB-435 [54], HBL-100 [54], and Hs578T (RI, unpublished data). Carnosol at 50 and 100 µM was shown to induce cell cycle blockade at the G2 phase [46]. This G2 block seems to be mediated through the overexpression of the CDK inhibitor p21 [38]. Indeed, p21 was shown to be upregulated only at concentrations that induced cell arrest [38]. Carnosol was also reported to cause G2 arrest in other cancer cell lines, such as MG-63 (osteosarcoma) [52], CaCo-2 (colon cancer) [60], and PC (prostate cancer) [37]. Interestingly, G2 block in PC3 cells was also associated with upregulation of p21. Thus, induction of G2 arrest is a common mechanism of action of carnosol against various types of cancer.

### 5.2. Carnosol Induces Programmed Cell Death (PCD) I and II in TNBC Cells via a ROS-Dependent Mechanism

Deregulation of apoptosis is implicated in several pathological conditions, including cancer [61]. Usually, cancer cells resist and escape apoptosis through the overexpression of antiapoptotic proteins and the repression of tumor suppressor genes, leading to uncontrolled proliferation and consequently to cancer development, progression, and treatment resistance [62,63,64].

**Figure 2:**
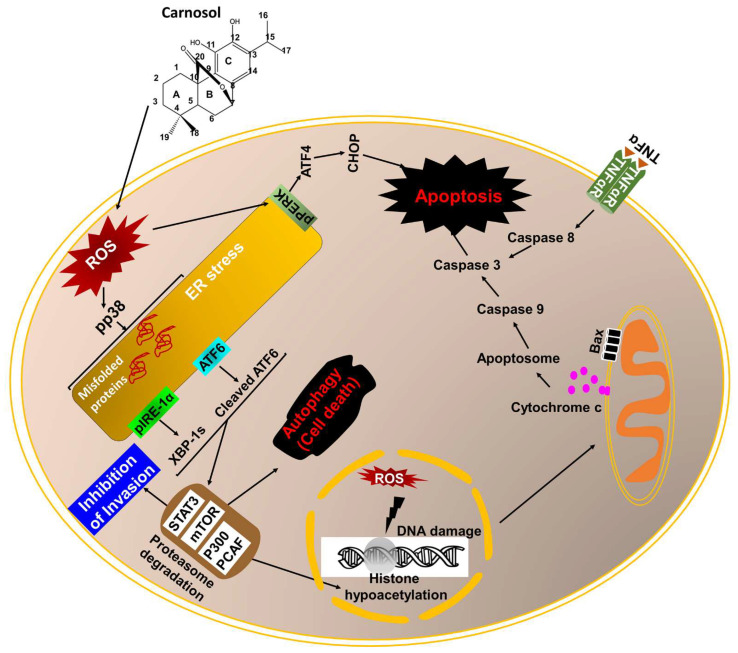
Mechanisms of action of carnosol against TNBC. Carnosol inhibits the cellular proliferation of TNBC by blocking the cell cycle at G2 phase via the upregulation of the cyclin-dependent kinase inhibitor p21. Carnosol stimulates ROS generation, leading to the activation of p38MAPK. Activated p38MAPK induces ER stress and activates the unfolded protein response (UPR) response pathway sensors IRE1α and ATF-6. Consequently, proteins such as mTOR, p300, PCAF, and STAT3 are targeted for proteasome degradation. The degradation of mTOR leads to the induction of excessive autophagy, which ultimately triggers autophagy-dependent cell death. ROS also activates, through a p38-independent mechanism, the PERK-ATF4-CHOP sensor, which might contribute to the activation of the intrinsic apoptosis pathway by inducing pro-apoptotic genes and downregulating the anti-apoptotic protein Bcl-2. CHOP could also contribute to the activation of the extrinsic pathway by upregulating the expression of death receptors. Excessive generation of ROS by prolonged exposure to carnosol leads to oxidative DNA damage that could also contribute to the activation of the programmed cell death pathways. Carnosol could also suppress TNBC migration and invasion through inhibition of the STAT3 pathway, leading to downregulation of MMP-9. Inhibition of the STAT3 pathway involves the targeting of STAT3 protein to proteasome degradation by the ROS-p38MAPK-ER stress cascade. Carnosol-mediated downregulation of p300 and PCAF protein levels, along with the resulting histone hypoacetylation, might also contribute to the inhibition of tumor growth and metastasis in triple-negative breast cancer.

Therefore, a better understanding of the molecular mechanisms underlying the resistance of TNBC to apoptosis will probably provide the foundations for developing targeted molecular therapies. TNBC is one of the most aggressive cancer types and is known to evade apoptosis and develop resistance to currently used therapeutic modalities, including chemotherapy and radiotherapy. Hence, inducing apoptosis in apoptosis-resistant cancer cells remains one of the key strategies in cancer treatment [65]. Al Dhaheri et al. showed that carnosol at 50 and 100 μM led to a concentration-dependent increase in the population of Annexin V-positive cells and accumulation of cleaved caspase 3 and cleaved PARP [38] in MDA-MB-231 treated for 24 h. Carnosol was shown to activate both the intrinsic and extrinsic apoptotic pathways in MDA-MB-231 cells. Indeed, active caspase 8 and caspase 9 were both detected in carnosol-treated cells [38] (Figure 2). Carnosol modulated the level of the anti-apoptotic/pro-apoptotic regulatory proteins Bcl-2/Bax and induced mitochondrial membrane potential depolarization, which are associated with the activation of the intrinsic pathway [38]. In addition, carnosol upregulated TNF-α (RI, unpublished data), a protein involved in the activation of the extrinsic pathway. More recently, Gonzalez–Cardenete et al. showed that novel semi-synthetic carnosol analogs inhibited cellular viability and induced apoptosis in MDA-MB-231 after 72 h with an IC_50_ between 1.3 and 18.7 μM [66]. However, the mechanism of action of these derivatives was not investigated. Still, these analogs could serve as a foundation for the development of novel anticancer compounds targeting TNBC.

Autophagy is a biological process that maintains cellular homeostasis by trafficking damaged or unwanted cellular components to the lysosome, thereby facilitating cellular survival. In the context of cancer, cancer cells can use autophagy as a cytoprotective mechanism to prevent the accumulation of cellular damages caused by chemotherapeutics, leading to the promotion of temporal survival and the development of chemoresistance [67]. Contrary to its cytoprotective role, autophagy is also known to induce cell death stimulated by excessive cellular stress, termed type II programmed cell death (PCDII) [68]. More importantly, cell death mediated by autophagy can involve the induction of apoptosis [69,70]. Consistently, inhibition of autophagy has been reported to reduce apoptosis in some tumors [71]. In this regard, both cell death mechanisms, apoptosis, and autophagy, may interplay with each other and share common mechanisms that drive the cellular death response. Carnosol, at a non-cytotoxic concentration (25 μM), was shown to induce autophagy in two TNBC cell lines, namely MDA-MB-231 [38,53] and Hs578-T (RI, unpublished data). Autophagy was confirmed by transmission electron microscopy (TEM), accumulation of lipidized LC3II, and decreased expression of p62 (SQSMT1) [38,53]. Interestingly, carnosol-mediated autophagy was independent of Beclin-1 [38]. Indeed, siRNA knockdown of Beclin-1 did not inhibit autophagy in carnosol-treated MDA-MB-231 cells. TEM showed that carnosol induced both mitophagy, characterized by swollen and damaged mitochondria, and reticulophagy [38,53], characterized by the accumulation of large numbers of swollen endoplasmic reticulum. Carnosol-induced autophagy seems to be mediated through downregulation of the mTOR pathway, a negative regulator of autophagy. Indeed, carnosol induced a dramatic decrease not only in the level of phosphorylated mTOR but also in the total mTOR protein [53] (Figure 2).

Considering that autophagy and apoptosis were both induced by carnosol in TNBC cells, Alsamri et al. examined the relationship between these two processes [53]. They found that carnosol-induced autophagy and apoptosis occurred independently of each other in TNBC. They showed that inhibition of autophagy by 3-methyladenine (3-MA) or chloroquine (CQ) only modestly rescued MDA-MB-231 from cell death. Similarly, inhibition of apoptosis by the pan-caspase inhibitor Z-VAD-FMK only had a slight effect on cellular viability. In contrast, a combination of autophagy and apoptosis inhibitors fully rescued carnosol-treated MDA-MB-231 cells from cell death [53]. This finding clearly shows that both programmed cell death mechanisms, apoptosis (PCDI) and autophagy (PCDII), were activated independently of each other in TNBC by carnosol. This was further confirmed by the accumulation of cleaved PARP and the persistence of the loss of mitochondrial membrane potential when autophagy was inhibited. The induction of autophagy and apoptosis in TNBC by carnosol was shown to be ROS-dependent, as inhibition of ROS by either tiron [38] or NAC [53] not only abolished the cytotoxic effect of carnosol on TNBC but also significantly reduced the level of LC3II (marker of autophagy) and cleaved PARP (marker of apoptosis) and restored the level of mTOR protein.

### 5.3. Carnosol Induces ER Stress via Activation of the p38-MAPK Pathway through a ROS-Dependent Mechanism

The unfolded protein response (UPR) is a highly regulated cascade system that is responsible for protein folding and trafficking and maintaining other cellular functions [72]. The UPR involves three key sensors located on the endoplasmic reticulum (ER) membrane, namely (*i*) protein kinase R-like ER kinase (PERK), (*ii*) transcription factor 6 (ATF6), and (*iii*) inositol-requiring enzyme 1α (IRE1α) [73]. Accumulating evidence suggests that the UPR signaling cascades are activated in response to ROS accumulation or oxidative triggers [74], which induce a cellular condition called ER stress, which is characterized by an accumulation of unfolded or misfolded proteins. ER stress leads to the activation of the UPR for the sake of restoring metabolic homeostasis, thus promoting cancer cell survival [75]. However, during severe ER stress, as a consequence of elevated or persistent oxidative stress, the UPR system triggers cancer cell death through activation of PCDI and/or PCDII [76]. Hence, finding or developing new drugs that stimulate ROS production and ultimately lead to UPR activation should be considered as an approach for new effective anticancer therapeutic approaches.

Recently, Alsamri et al. showed that carnosol triggers the activation of the three UPR sensors (PERK, IRE1α, and ATF-6) in MDA-MB-231 [53] and Hs578T cells (RI, unpublished data). Indeed, carnosol induced the phosphorylation of IRE1α and EIF2 α, upregulated the protein levels of XBP-1s, cleaved ATF6, ATF4, and CHOP, and downregulated PDI and Ero-1 α, which are enzymes that promote proper protein folding in the ER [53]. These biochemical data were in agreement with TEM data that showed dilated and multilamellar ER [53]. The activation of UPR sensors by carnosol seems to depend solely on ROS accumulation (Figure 2). Indeed, NAC pretreatment was sufficient to completely abolish the activation of the three UPR sensors by carnosol and restore the normal levels of PDI and Ero-1 and, thus, the protein folding function of the ER [53].

The p38 MAPK pathway translates extracellular signals to the intracellular machinery that regulates various cellular processes, such as cell cycle, cell death, differentiation, and senescence. Depending on the kinetics of activation, downstream signaling pathways are consequently activated [77,78,79]. Several studies showed that p38 plays a dual role in the UPR cascade [80,81]. p38 can be activated as a consequence of IRE-1α and PERK oligomerization [81,82]. On the other hand, prolonged activation of p38 induces ER stress and activates the UPR response pathway by regulating the expression of UPR components such as CHOP [39], ATF6 [39], and XBP-1s [83]. Moreover, studies showed that in the presence of ER stress, p38 can promote cell death through the induction of autophagy and/or apoptosis in various types of cancer, depending on the type and duration of the stimulus. Carnosol was shown to activate p38MAPK in MDA-MB-231 cells in a ROS-dependent manner [53] (Figure 2). Time course analysis revealed that the phosphorylation of p38MAPK occurred concomitantly with ROS generation, i.e., 1 h post-carnosol treatment [53]. However, the activation of p38MAPK was shown to be dependent on ROS since NAC pretreatment was sufficient to block the activation of p38MAPK [53]. Interestingly, p38MAPK activation was the earliest event induced by carnosol; it preceded the activation of the UPR pathways, induction of autophagy, and activation of the apoptotic pathway, which occurred at 3, 6, and 24 h post-treatment, respectively. Thus, p38 seems to play a central role in the anti-TNBC effect of carnosol. Indeed, chemical inhibition of p38MAPK activation by either of the two p38 inhibitors, SB202190 or SB203580, was sufficient to partially reduce the cytotoxic effect of carnosol, as well as efficiently block the activation of the IRE-1α and ATF6 pathways and induction of autophagy [53] (Figure 2). Carnosol-mediated apoptosis, however, seems to occur independently of p38MAPK. Considering the fact that carnosol induces sustained DNA damage [38] in addition to protein misfolding [53], we hypothesize that the activation of apoptosis is a secondary response to excessive cellular damage due to prolonged exposure to carnosol.

### 5.4. Carnosol Inhibits p300 Acetyl Transferase

Acetylation of histones and non-histone proteins is the most abundant modification that is involved in various key cellular processes, such as cellular proliferation, cell-cycle progression, differentiation, apoptosis, dosage compensation, hormonal signaling, gene transcription, DNA damage repair, protein folding, autophagy, and metabolism [84,85,86,87,88]. The dynamic and reversible acetylation of histone and non-histone proteins is a major epigenetic mechanism that regulates gene transcription, and deregulation of this process has been implicated in a wide variety of human diseases, including cancer [89,90,91,92,93,94,95]. Thus, proteins involved in the regulation of acetylation status are considered an attractive therapeutic target. Acetylation of histones and non-histone proteins is regulated by a class of enzyme family members, the histone acetyltransferases (HATs) [96,97]. One such enzyme, p300 HAT, has been associated with tumor development and progression [98,99,100,101]. Deregulation of HAT activity is particularly linked to the formation and progression of different types of cancer, including breast cancer [102,103]. Indeed, p300 was reported to be upregulated in invasive breast cancer [104,105]. Carnosol was reported to induce a concentration-dependent decrease in the overall expression of histone H3 and H4 in MDA-MB-231 and Hs578T cells [56]. In silico docking analysis revealed that carnosol can bind to the acetyl-CoA binding pocket of the catalytic domain of p300, thus potentially blocking its activity [56]. This was confirmed by an in vitro HAT assay in which carnosol was able to completely inhibit the ability of p300 to bind to histone H3 and histone H3K56, a preferred substrate of p300. This inhibition could only be partially relieved by a large excess of acetyl-CoA (400 μM), thus suggesting competitive inhibition of p300 by carnosol [56]. Given that p300 was targeted for proteasomal degradation by carnosol in TNBC, it is hard to appreciate the extent of this specific inhibition on the anti-TNBC of carnosol. It is hence worth testing the effect of p300 inhibition in other cancer cell types where carnosol-mediated p300 degradation does not occur.

### 5.5. Carnosol, a Potential Targeted Protein Degradation Molecule, Targets Key Proteins Regulating Cancer Growth and Metastasis through a p38MAPK-Dependent Mechanism

Targeted protein degradation (TPD) via the proteasome via stimulation of the ubiquitin proteasome system is gaining attention as a novel and promising anticancer therapeutic approach [106]. There are at least ten TPD molecules in clinical trials against various types of cancer [107]. For example, ARV-471, now in a phase II clinical trial, was shown to specifically target the ER, a regulator of breast cancer pathogenesis [107]. KT-333 is another PTD molecule in a phase I clinical trial that was shown to specifically target STAT3 in multiple xenograft mouse models [107]. Interestingly, carnosol was reported to possess PTD activity against several key regulator proteins, including STAT3, mTORC1, p300, and PCAF. Indeed, carnosol targeted these proteins for proteasomal degradation. It is noteworthy to mention that carnosol also induced an increased level of overall protein ubiquitination in MDA-MB-231 cells. Protein polyubiquitination and degradation of STAT3, mTORC1, p300, and PCAF occurred through a ROS-dependent mechanism, as NAC pretreatment completely reversed this effect [50,53,56]. Interestingly, the targeting of these four proteins for proteasomal degradation is dependent upon the activation of p38MAPK (Figure 2). Indeed, chemical inhibition of p38MAPK by SB202190 not only reduced the level of protein polyubiquitination in MDA-MB-231 but also inhibited the degradation of STAT3, mTORC1, p300, and PCAF [53]. Hence, the mechanism through which p38MAPK targets STAT3, mTORC1, p300, and PCAF for proteasomal degradation warrants further investigation.

### 5.6. Carnosol Inhibits Tumor Growth and Metastasis in TNBC

Cancer metastasis is a complex process involving several steps. Invasion is a critical step in cancer metastasis that involves proteolytic degradation of the extracellular matrix. It has been reported that elevated levels of matrix metalloproteinases (MMPs) are linked to the aggressiveness and metastatic potential of breast cancer [108,109,110]. Inhibiting the expression or activity of these MMPs is considered a potential therapeutic approach against breast cancer. Alsamri et al. reported that carnosol significantly and efficiently inhibited the migration and invasion potential of TNBC cells both in vitro and in vivo [50]. It also significantly inhibited tumor growth of MDA-MB-231 in vivo, as demonstrated with the chick embryo tumor growth and metastasis assay [50]. The anti-metastatic activity of carnosol involved the inhibition of both the expression and activity of MMP-9, as demonstrated with gelatin zymography, ELISA, and RT-PCR assays [50]. Similar findings were reported when carnosol was used against the highly metastatic mouse melanoma cell line B16/F10 [111]. Thus, inhibition of the expression and activity of MMP-9 seems to be a general mechanism underlying carnosol-mediated inhibition of cancer cell invasion. Interestingly, inhibition of the invasion of TNBC cells involved inhibition of the STAT3 signaling pathway, which is known to regulate the expression of MMP-9. The inhibition of the STAT3 pathway by carnosol was mediated through the ROS-dependent targeting of STAT3 protein to proteasomal degradation in TNBC MDA-MB-231 and Hs578T cells, and also in non-TNBC MCF-7 and T47D breast cancer cells [50]. While screening for potential new STAT3 inhibitors, Yanagimichi et al. reported that carnosol induced a dramatic decrease in the level of STAT 3 protein in HepG2 hepatocellular carcinoma cells [112]. Intriguingly, in HCT116 colon cancer cells, although carnosol inhibited STAT3 phosphorylation, it did not affect the level of total cellular STAT3 [41,51]. It is also noteworthy to mention that inhibition of MMP-9 expression and, thus, the inhibition of invasion of the highly metastatic mouse melanoma B16/F10 cells occurred independently of STAT3 and was attributed to the inhibition of the ERK 1/2, AKT, p38, JNK, and NF-kB signaling pathways [111]. Hence, the cell-type-specific effect of carnosol on STAT3 warrants further investigation.

### 5.7. Synergetic Anticancer Effects of Carnosol in an In Vitro Combination Assay

Despite the increasing interest in the anticancer effect of carnosol, specifically with respect to cancer initiation and progression, few studies have shown the efficacy of using diterpenoids in combination with other phytochemicals or chemotherapeutic agents [113,114,115]. Vergara et al. investigated the cytotoxic effect of carnosol alone and in combination with other phytochemicals or commonly used chemotherapeutic drugs on several human cancer cell lines, including TNBC (HBL-100, MDA-231, and MDA-435) cells [54]. Vergara et al. treated TNBC cells with carnosol (50 μM) and curcumin (70 μM) for 4 h and assessed their potential synergetic effects using western blotting. Combination therapy reduced the expression of Bcl-2, cyclin D1, and survival, and increased the expression of p27 more efficiently than mono-treatment. These findings highlight the promising synergetic potential of carnosol and curcumin against TNBC by decreasing viability and inducing apoptosis and cell cycle blockade [54]. These findings warrant further investigation of the effect of the combination of carnosol with currently used anticancer chemotherapeutic drugs against TNBC.

## 6. Conclusions

Carnosol, a phytochemical abundant in rosemary and sage extracts, has long been recognized for its many biological and medicinal properties, including antioxidant, antimicrobial, anti-inflammatory, neuroprotective, and anticancer effects. The anticancer effect of carnosol against various types of cancer has gained much interest in the recent past. Interestingly, carnosol has proven its effectiveness against TNBC through the modulation of several biochemical pathways (Figure 2). Indeed, carnosol exerts its effect against TNBC by targeting and modulating several cancer hallmarks. We should take advantage of the fact that carnosol can be isolated from widely abundant perennial plants, and therefore a constant supply of this agent can be secured for further studies. Further, increasing evidence from recent preclinical studies strongly suggests that carnosol is a potential promising chemopreventive and chemotherapeutic agent against TNBC. Therefore, more focus should be given to in vivo studies with carnosol, which are still dramatically lacking.

## Figures and Tables

**Table 1:** Summary of the anti-TNBC effect of carnosol.

Cell Line	Carnosol		Experimental Model			Targeted Proteins		Reference
	Alone	In Combination	In Vitro	In Vivo	In Silico	Upregulated	Downregulated	
HBL-100 MDA-231 MDA-435	12.5–200 μM; 4 h, 24 h, 48 h and 72 h	Carnosol 50 μM + Curcumin 70 μM; 4 h	✓	-	-	p27	Cyclin D1 Bcl2 Survivin	[54]
MDA-MB-231	25–100 μM; 24 h and 48 h	-	✓	-	-	p21/WAF1 Bax LC3II pERK1/2 γH2AX	p27 PARP Cleaved Caspases 3,8,9 Bcl2 p62 (SQSTM1)	[38]
MDA-MB-231	EC50 < 9 μM	-	✓	-	-			[55]
MDA-MB-231 Hs578T	25–100 μM	-	✓	✓	-		MMP-9 STAT3	[50]
MDA-MB-231 Hs578T	25–100 μM; 24 h	-	✓	-	✓		Ac-H3K14 Ac-H3K56 Ac-H4K9 Ac-H4K16 Ac-H4K5 P300 PCAF	[56]
MDA-MB-231 Hs578T	50 and 100 μM; 24 h	-	✓	-	-	PARP LC3 I/II IRE1α XBP-1s Cl. ATF6 eIF2α ATF4 CHOP pP38 Ubiquitination	mTOR PDI Ero1α PCAF STAT3	[53]

## Data Availability

Not applicable.
